# General and Specific Cytotoxicity of Chimeric Antisense Oligonucleotides in Bacterial Cells and Human Cell Lines

**DOI:** 10.3390/antibiotics13020122

**Published:** 2024-01-26

**Authors:** Katya B. Popova, Robert Penchovsky

**Affiliations:** 1 Laboratory of Synthetic Biology and Bioinformatics, Faculty of Biology, Sofia University “St. Kliment Ohridski”, 8 Dragan Tzankov Blvd., 1164 Sofia, Bulgaria; 2 Institute for Nuclear Research and Nuclear Energy, Bulgarian Academy of Sciences, 1784 Sofia, Bulgaria

**Keywords:** antisense oligonucleotides, general toxicity, specific toxicity, off-target, *Staphylococcus aureus*, human cell lines

## Abstract

In the last two decades, antisense oligonucleotide technology has emerged as a promising approach to tackling various healthcare issues and diseases, such as antimicrobial resistance, cancer, and neurodegenerative diseases. Despite the numerous improvements in the structure and modifications of the antisense oligonucleotides (ASOs), there are still specific problems with their clinical efficacy and preclinical cytotoxicity results. To better understand the effects of the ASOs in this paper, we conducted many MTT assays to assess the general and specific cytotoxicity of four new chimeric ASOs in bacterial cells and human cell lines. We demonstrate the absence of inhibitory activity in the human pathogenic bacteria *Staphylococcus aureus* by non-specific ASOs. The pVEC-ASO1 and pVEC-ASO2 are designed to have no specific targets in *S. aureus*. They have only partial hybridization to the guanylate kinase mRNA. The pVEC-ASO3 targets UBA2 mRNA, a hallmark cancer pathology in MYC-driven cancer, while pVEC-ASO4 has no complementary sequences. We discovered some cytotoxicity of the non-specific ASOs in healthy and cancer human cell lines. The results are compared with two other ASOs, targeting specific mRNA in cancer cells. All ASOs are delivered into the cell via the cell-penetrating oligopeptide pVEC, which is attached to them. We draw a good correlation between the thermodynamic stability of ASO/target RNA and the toxicity effect in human cell lines. The data obtained signify the importance of thorough bioinformatic analysis and high specificity in designing and developing novel ASOs for safer therapeutic agents in clinical practice.

## 1. Introduction

The need for the faster development of newer and improved drugs has increased exponentially [1]. Most currently available drugs target proteins or act as proteins [2]. Other drug research and development (R&D) has emerged in recent decades based on antisense oligonucleotide technology. This technology targets RNAs instead of proteins using antisense oligonucleotides (ASOs). The ASOs are single-stranded (ss) oligonucleotides, typically between 18nt and 25nt in length [3,4]. They can inhibit gene expression by binding complementarily to a target RNA [5]. ASOs achieve that by inhibiting the RNA translation via steric blockage or by various RNase-mediated cleavage and degradation of the target RNA, such as RNases H and P [6,7,8,9].

The ASOs need specific chemical modifications to be more stable and resilient to nuclease degradation without off-target hybridization. These modifications can divide the ASOs into three generations. First-generation modifications of ASOs have phosphorothioate (PS) bonds. They are characterized by substituting one of the non-bridging oxygen atoms with a sulfur one in the phosphodiester bond. Second-generation ASO modifications include the 2′-alkyl modifications of the ribose, usually 2′-O-methyl or 2′-O-methoxyethyl. They protect the second-generation ASOs from nucleases better than the first-generation PS modifications and improve their site-specific binding and stability. However, compared to the previous generation, they cannot activate the RNase H-mediated cleavage of targeted RNA and, therefore, work under a single-turnover mode in contrast to the multi-turnover mode of the PS-ASOs. Thus, chimeric (gapmer) ASOs are usually designed with first- and second-generation modifications. The sequence’s central “gap” part has PS modifications to activate RNase H-mediated cleavage of the targeted RNA. At the same time, 2′-alkyl modifications flank the middle part, which makes the chimeric structure more resistant to nucleases. Third-generation modifications are more diverse and mainly involve the furanose ring. This modification includes adding a bridge between 2’-oxygen and 4’-carbon in the locked nucleic acid (LNA), further improving the ASO/RNA hybridization specificity. Overall, making these modifications and synthesizing ASOs are relatively cheap. Therefore, antisense oligonucleotide technology provides a particular and valuable method for controlling the transcriptome and the proteome.

The recent progress toward clinical applications and approvals of ASO-based drugs is encouraging. Many applications have been found in treating various diseases associated with RNA dysregulation and mutations. For example, inflammatory bowel diseases, such as Crohn’s, and ulcerative colitis, neurodegenerative diseases, including Huntington’s, amyotrophic lateral sclerosis, and many types of cancer, are considered to be treated with ASOs [10,11,12,13,14,15,16,17,18,19,20,21,22,23,24,25,26,27,28]. Furthermore, there are already six FDA-approved ASO-based drugs for the treatment of patients, including fomivirsen for the treatment of cytomegalovirus retinitis in 1998, pegaptanib for the treatment of neovascular age-related macular degeneration in 2004, mipomersen for familial hypercholesterolemia in 2013, eteplirsen for Duchenne muscular dystrophy in 2016, nusinersen for spinal muscular atrophy in 2016, and volanesorsen for familial chylomicronemia syndrome [29,30]. In addition, another ASO-based drug, called milasen, was approved for a child-targeted therapy of a rare neurodegenerative disease, such as neuronal ceroid lipofuscinosis 7 [31].

Moreover, sequence-specific RNAs are present only in bacteria or viruses, making them the perfect target for antibacterial [7,8,9] and antiviral drugs based on antisense oligonucleotide technology [32,33,34,35,36,37,38,39,40]. We recently developed a rational approach for designing antibacterial ASOs that target riboswitches [5]. Our method is completely rational, including criteria for evaluating the mRNA targets and the design of ASOs. These criteria have been proven to be accurate in four instances [6,7,8,9]. Therefore, it is important to evaluate the toxicity of ASOs for near-future drug development.

Our research is focused on designing new chimeric ASOs and evaluating their non-specific and specific toxicity in pro- and eukaryotes. The toxicity of three ASOs is assessed in the human pathogenic bacteria *Staphylococcus aureus*, one of the most frequent causes of hospital-acquired infections in patients [41]. The specific and non-specific toxicity of four ASOs is also assessed in a human regular cell line (Lep3) and cancer cell lines (MDA-MB-231 and HT29).

In this paper, we first established a good correlation between the thermodynamic stability of ASO/target RNA and the toxicity effect in human cell lines. The findings are very important for further establishing rational criteria for designing ASOs with embedded reduced non-specific toxicity.

## 2. Materials and Methods

### 2.1. Materials

**Figure 1:**
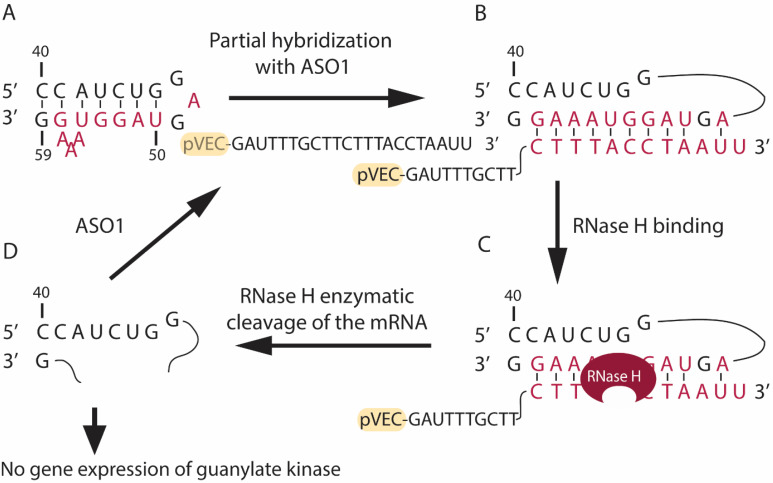
Partial hybridization in the guanylate kinase mRNA of *S. aureus* (strain ATCC 25923) with the designed pVEC-ASO1. (**A**) The region of the mRNA responsible for guanylate kinase synthesis in *S. aureus* (strain ATCC 25923) is shown. The sequence that will partially hybridize with pVEC-ASO1 is marked in red. (**B**) The pVEC-ASO1 can enter the bacterial cell due to its attached cell-penetrating peptide (pVEC). Next, pVEC-ASO1 hybridizes with the targeted mRNA sequence, forming a double-stranded RNA/DNA duplex (red) with a free energy of the thermodynamic ensemble of −12.09 kcal/mol. (**C**) RNAse H recognizes the site of the RNA/DNA duplex and its PS modifications. Thus, it specifically hydrolyzes the phosphodiester bonds in the formed duplex. (**D**) The enzymatic hydrolysis of the mRNA leads to the absence of gene expression of the essential guanylate kinase in *S. aureus*.

**Figure 2:**
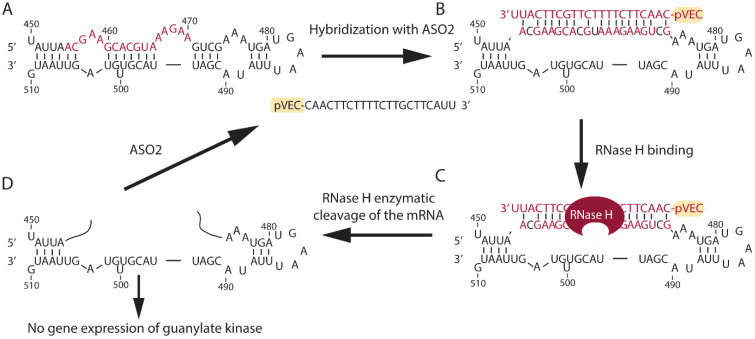
Partial hybridization in the guanylate kinase mRNA of *S. aureus* (strain ATCC 25923) with the designed pVEC-ASO2. (**A**) The region of the mRNA responsible for guanylate kinase synthesis in *S. aureus* (strain ATCC 25923) is shown. The sequence that will partially hybridize with pVEC-ASO2 is marked in red. (**B**) The pVEC-ASO2 can enter the bacterial cell due to its attached cell-penetrating peptide (pVEC). Then, the pVEC-ASO2 hybridizes with the targeted mRNA sequence and forms a double-stranded RNA/DNA duplex with a free energy of the thermodynamic ensemble of −9.38 kcal/mol (red). (**C**) RNAse H recognizes the site of the RNA/DNA duplex and its PS modifications. Thus, it specifically hydrolyzes the phosphodiester bonds in the formed duplex. (**D**) The enzymatic hydrolysis of the mRNA leads to the absence of gene expression of the essential guanylate kinase in *S. aureus*.

**Figure 3:**
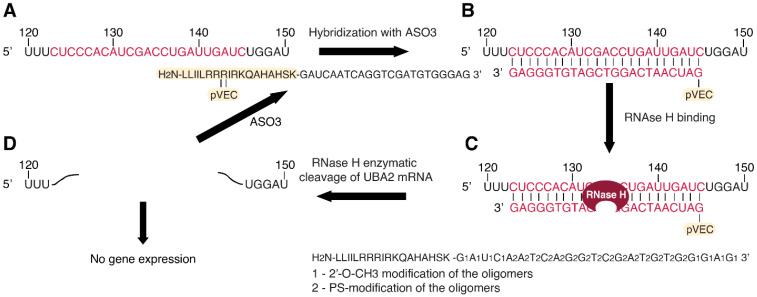
Specific targeting in the Homo sapiens ubiquitin-like modifier activating enzyme 2 (UBA2) mRNA with the designed pVEC-ASO3. (**A**) Region of the UBA2 mRNA is shown. The sequence we are targeting with pVEC-ASO3 is marked in red. (**B**) pVEC-ASO3 is designed to enter the bacterial cell due to its attached cell-penetrating peptide (pVEC). Hence, pVEC-ASO3 hybridizes to the targeted mRNA sequence, forming a double-stranded RNA-DNA duplex (red) with a free energy of −29.77 kcal/mol. (**C**) RNase H recognizes the site of the RNA-DNA duplex and its PS modifications. Thus, it specifically hydrolyzes the phosphodiester bonds in the formed duplex. (**D**) The enzymatic hydrolysis of the mRNA leads to the absence of gene expression of the UBA2 mRNA.

Four antisense oligonucleotides named pVEC-ASO1 (Figure 1), pVEC-ASO2 (Figure 2), pVEC-ASO3 (Figure 3), and pVEC-ASO4 were purchased from Gene Link, Inc., Elmsford, NY, USA (Table 1). All ASOs were attached to the cell-penetrating oligopeptide (CPP) pVEC built of 18 Amino Acid Residues (AARs) with a molecular mass of 2209.47 Da. Our study used the human pathogen *Staphylococcus aureus* strain ATCC 25923 obtained from the German Collection of Microorganisms and Cell Cultures (DSMZ) GmbH, Braunschweig, Germany.

Cell line experiments were conducted with three continuous cell cultures: (1) MDA-MB-231 from human breast adenocarcinoma, (2) HT29 from human colorectal adenocarcinoma, and (3) Lep3 non-tumor cell line from 3-month human embryos. Luria–Bertani (LB) medium and LB agar were prepared with chemicals purchased from Oxoid LTD (Basingstoke, UK) [42], while the plates were from Corning Incorporated (New York, NY, USA). The Dulbecco′s Modified Eagle′s Medium (DMEM) and fetal bovine serum were bought from Gibco-Invitrogen (New York, NY, USA) [42]. We also ordered trypan blue and dimethyl sulfoxide (DMSO) from AppliChem, Germany. All the rest of the chemicals were purchased from local suppliers.

### 2.2. Methods

#### 2.2.1. Bioinformatical Analyses

We used the Genbank of NCBI and KEGG PATHWAY databases to search for suitable target sequences. We conducted BLAST (Basic Local Alignment Search Tool) analysis to avoid off-target hybridization by comparing similar nucleic sequences of the chosen ASO targets. We also used ClustalX (2.0) and Clustal W (1.83) from the EMBOSS to align all of the close similarities of the sequences in multiple alignments. We used the partition function for the RNA folding secondary of the targeted sequences with the Vienna RNAcofold web server. Therefore, the thermodynamic ensemble of RNA/DNA region formed by ASO and the target RNAs was computed by the RNAcofold applet of Vienna RNA folding web server at http://rna.tbi.univie.ac.at/cgi-bin/RNAWebSuite/RNAcofold.cgi (accessed on 11 January 2024). The ASOs were designed using databases, including NCBI (https://www.ncbi.nlm.nih.gov/, accessed on 11 January 2024), KEGG: Kyoto Encyclopedia of Genes and Genomes (https://www.genome.jp/kegg/, accessed on 11 January 2024), ExPASy bioinformatics resource portal (https://www.expasy.org/, accessed on 11 January 2024) and Rfam 13.0 (http://rfam.xfam.org/, accessed on 11 January 2024).

#### 2.2.2. Bacterial Cultivation

We used two media for bacterial cultivation, Luria–Bertani (LB) broth and LB agar. Both were prepared according to a protocol described by Sambrook et al. [43]. *Staphylococcus aureus* ATCC 25923 was used in this study. They were stored in a 20% glycerol stock at −80 °C. We revived and cultivated them overnight in LB medium at 37 °C in a Memmert incubator. When the bacteria were in the exponential phase at an Optical Density (OD) of 0.7–0.8, we made a 1:100 dilution of the inoculate with LB medium. We used different concentrations of pVEC-ASOs (3 repeats for each) to treat the diluted bacteria in cuvettes suitable for the spectrophotometer. The bacterial growth was measured every 30 min at 600 nm wavelength by Pharmacia Biotech Ultrospec 100E UV/Visible spectrophotometer. All tested cuvettes were cultured at 37 °C in an environmental shaker incubator ES-20 Biosan.

#### 2.2.3. Cell Cultures and MTT Assay

All cell lines were grown in D-MEM supplemented with 5–10% fetal bovine serum (FBS), 100 U/mL penicillin, and 100 μg/mL streptomycin. They were seeded in 96-well microplates and kept in a humidified incubator (Thermo Scientific, HEPA Class 100, Waltham, MA, USA) at 37 °C under 5% CO_2_ in the air. The cell cultures were treated with the designed pVEC-ASOs or with pVEC alone. They were cultured in the humidified incubator for 72 h at 37 °C. Next, MTT assays were performed as described by Mosmann et al. [44]. The assays were measured on an ELISA reader (TECAN, SunriseTM, Grodig/Salzburg, Austria).

## 3. Results

### 3.1. Design of Antisense Oligonucleotides

We designed four new ASOs for our experiments (Table 1). We used two types of modifications of our ASOs, such as 2’-O-methylation and PS modifications. The 2’-O-methylation modifications were applied to the two wings of the ASOs (Table 1). The PS modifications were used in the ASO central (gap) part. In addition, all ASOs were attached in their 5’-ends to the C-terminus of the cell-penetrating oligopeptide pVEC. Among the tested pVEC-ASOs, pVEC-ASO1 and pVEC-ASO2 partially hybridized only with their target, pVEC-ASO3 had complete hybridization with its target, and pVEC-ASO4 did not hybridize with any sequence.

pVEC-ASO1 (Figure 1) and pVEC-ASO2 (Figure 2) are 22 nt long and are not entirely complementary to the guanylate kinase mRNA in the human pathogen *S. aureus* (Figure S1). The pVEC-ASO1 forms a double-stranded RNA/DNA region with a free energy of the thermodynamic ensemble of −12.09 kcal/mol (Figure 1). In comparison, pVEC-ASO2 forms a double-stranded RNA/DNA region with a free energy of the thermodynamic ensemble of −9.33 kcal/mol (Figure 2). The pVEC-ASO3 is 23nt long and designed to target mRNA, forming a double-stranded RNA/DNA region with a free energy of the thermodynamic ensemble of −29.77 kcal/mol (Figure 3) with the Homo sapiens UBA2 gene (Figure S2). The pVEC-ASO3 targets a region between 123nt and 145nt of the UBA2 mRNA (Figure 3A). It hybridizes when entering human cells, forming a chimeric DNA-RNA duplex (Figure 3B). RNase H can recognize the ASO3/RNA hybrid (Figure 3C). Thus, RNase H-mediated cleavage of the mRNA is activated. As a result, a small region of the DNA/RNA duplex is cleaved (Figure 3D). On the contrary, pVEC-ASO3 can form other DNA/RNA hybridizations and RNase H-mediated cleavage, therefore working under multi-turnover conditions. pVEC-ASO4 is an 11nt-long chimeric ASO designed to have no complementary sequences in either *S. aureus* or the human genomes.

### 3.2. Bioinformatics Analyses

The BLAST analyses of pVEC-ASO1 (Figure S3) in the GeneBank database demonstrated that the highest sequence similarity between its target and the bacterial genomes is 68% (Figure S3A). Meanwhile, compared to the human genome, pVEC-ASO1′s targeted region has a maximum of 90% query coverage in two predicted RNAs (Figure S3B). Similarly, BLAST analyses were performed for pVEC-ASO2′s complementary sequence in the GeneBank database (Figure S4). The results demonstrated that the highest sequence similarity between its target and a bacterial genome is 86% (Figure S4A). At the same time, the analysis for similarities in the human genome demonstrated that pVEC-ASO2′s targeted region has a maximum of 72% query coverage (at 100% identity), with three annotated and six predicted human RNAs (Figure S4B) and a maximum of 95% query cover with the BRAF proto-oncogene.

A similar analysis was performed for pVEC-ASO3′s targeted region in the GeneBank database (Figure S5). The RNAref results in bacteria demonstrated the highest query cover of 95% with a symbiont of aphids, followed by two other bacteria, with 91% and 86% query cover, which is also not part of the human microbiome (Figure S5A). The same analysis in the human genome demonstrated complete similarity only with the targeted UBA2 mRNA and two predicted transcript variants (X6 and X7, Figure S5B). The next highest similarity, not with another predicted transcript variant of the target, was observed in transmembrane protein 170A mRNA and its various transcript variants at barely 65% query cover. On the other hand, pVEC-ASO4′s hypothetical targeted sequence demonstrated no significant similarity after setting the RNAref analysis for all genomes (Figure S6).

### 3.3. Cell Growth Inhibition by Antisense Oligonucleotides

**Figure 4:**
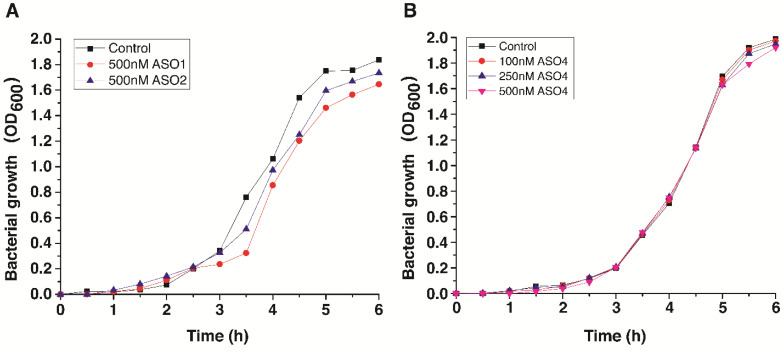
A growth curve of *S. aureus* ATCC 25923 strain cultured in LB medium and treated with pVEC-ASO1, pVEC-ASO2, and pVEC-ASO4. (**A**) Bacteria were treated with 500 nM pVEC-ASO1 (red circle line) and 500 nM pVEC-ASO2 (blue triangle line). The treated bacteria are compared to the control with untreated bacteria (black square line). (**B**) Bacteria were treated with 100 nM pVEC-ASO4 (red circle line), 250 nM pVEC-ASO4 (blue triangle line), and 500 nM pVEC-ASO4 (pink upside-down triangle). The treated bacteria are compared to the control with untreated bacteria (black square line). All the measurements were assessed in cuvettes by spectrophotometer at 600 nm wave.

A total of four antisense oligonucleotides and one cell-penetrating peptide were tested in bacteria or different human cell lines. First, we probed the antibacterial activity of pVEC-ASO1, pVEC-ASO2, and pVEC-ASO4 in *S. aureus* ATCC 25923 (Figure 4). The experimental results showed low levels of inhibition when we treated *S. aureus* with pVEC-ASO1 and pVEC-ASO2 separately. Control samples containing only *S. aureus* in LB medium reached the log phase after only 1 h (Figure 4A). The growth rate decreased 5 h after treatment, reaching the stationary phase at 1.75 OD. However, the test revealed that applying 500 nM of pVEC-ASO1 has an approximately 33% inhibitory effect (0.24 OD) 3 h after treatment of the bacteria. The inhibition decreased to around 20% for the next 2 h (the exception is the increase in the values to approximately 57% inhibition 3.5 h after treatment). Furthermore, only 12% inhibition (1.67 OD) was observed 6 h after treatment. On the other hand, pVEC-ASO2 showed overall lower inhibition of *S. aureus*. The maximum inhibition was reached 4.5 h after the treatment at values of approximately 19% (1.25 OD), which decreased to 8% (1.74 OD) 6 h after treatment.

Similar tests were conducted with pVEC-ASO4 using the spectrophotometer (Figure 4B). For this research, *S. aureus* was treated with three different concentrations of pVEC-ASO4, including 100 nM, 250 nM, and 500 nM. The results were compared with the controls and showed no evidence, suggesting the presence of pVEC-ASO4-induced bacteria inhibition at any concentration. Furthermore, we performed analogical tests with the cell-penetrating peptide pVEC to determine its role in the experimental data. We treated *S. aureus* with different concentrations of pVEC (250 nM, 500 nM, 1000 nM, and 2000 nM), and the obtained data suggested no significant inhibition caused by pVEC in the bacteria in vivo (Figure S7).

**Figure 5:**
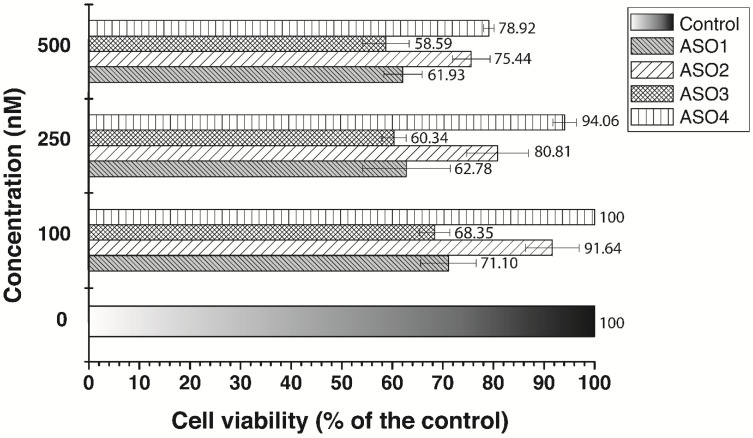
MTT viability assay. The viability of the adenocarcinoma MDA-MB-231 cell line was measured at 540/620 nm. Results are expressed as the percentage of the cell viability relative to the control. Data are presented as the mean ± S.D. (standard deviation) after exposure for 72 h to different concentrations of the ASOs. All tested ASOs contain pVEC attached in their 5’-end, and the obtained data is not from cell lines treated with ASO without their CPP.

**Figure 6:**
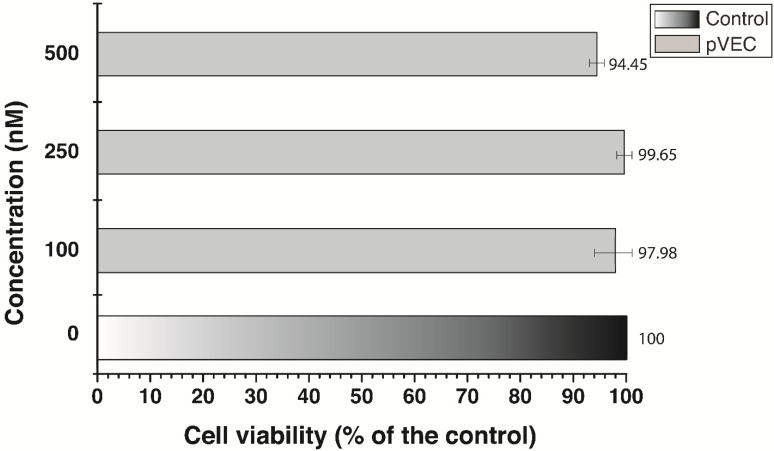
MTT viability assays of the adenocarcinoma MDA-MB-231 cell line were measured at 540/620 nm. Results are expressed as the percentage of the cell viability relative to the control. Data are presented as the mean ± S.D. after exposure for 72 h to different concentrations of the cell-penetrating peptide pVEC.

Two MTT assays were conducted in a human adenocarcinoma cell line MDA-MB-231 (Figure 5 and Figure 6). All four pVEC-ASOs were tested in three different concentrations, such as 100 nM, 250 nM, and 500 nM. The results were measured 72 h after treatment at the 540/620 nm wavelength. Furthermore, all obtained results showed more than 50% survival of the MDA-MB-231 cells. Our results demonstrated that at the lowest applied concentration (100 nM), there is no inhibition of the cells treated with pVEC-ASO4 (Figure 5). The highest level of inhibition was observed in cells treated with pVEC-ASO3, with 68.35% cell viability. Our findings at the highest concentration were similar to those previously mentioned. Adenocarcinoma cells treated with pVEC-ASO3 demonstrated the lowest cell viability of 58.59%, while the same treated with ASO4 had the highest viability of 78.92%. Based on these findings, we calculated an MIC_50_ = 604 nM for pVEC-ASO3. On the other hand, the MTT assay with the MDA-MB-231 treated with pVEC only revealed no significant inhibition at concentrations of 100 nM and 250 nM (Figure 6). It had only a 5.6% inhibitory effect at the highest applied concentration of 500 nM pVEC. These results suggest two things. First, pVEC has no role in the inhibitory effects of pVEC-ASO1, pVEC-ASO2, pVEC-ASO3, and pVEC-ASO4. Second, pVEC-ASO3, followed by pVEC-ASO1, has the highest potential as an anti-tumor agent among all tested pVEC-ASOs due to the observed cytotoxicity in the MDA-MB-231 cell line.

**Figure 7:**
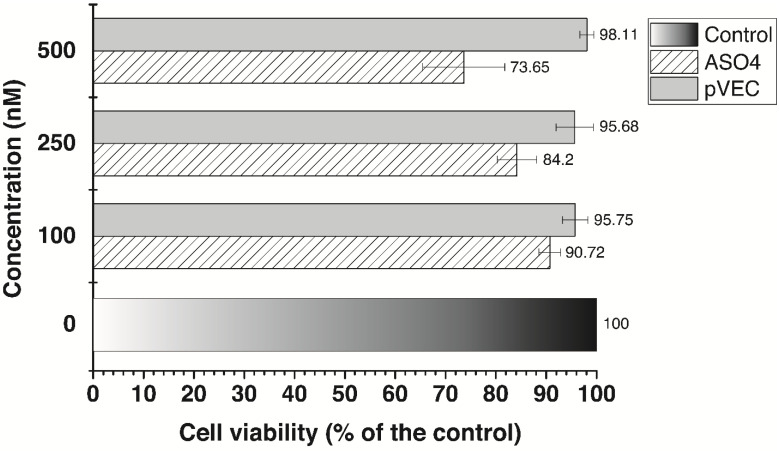
MTT viability assay. The viability of the colorectal adenocarcinoma HT29 cell line was measured at 540/620 nm. Results are expressed as the percentage of the cell viability relative to the control. Data are presented as the mean ±S.D. after exposure for 72 h to different concentrations of ASO4 or the cell-penetrating peptide pVEC. pVEC-ASO4 contains pVEC attached in its 5’-end, and the obtained data is not from cell lines treated with ASO4 without the CPP.

A similar experimental pattern was performed in colorectal adenocarcinoma cell line HT29 using the MTT assay (Figure 7). The concentrations used in this study are the same as in the previous MTT assays, including 100 nM, 250 nM, and 500 nM. Measurements were taken 72 h after the treatment at 540/620 nm wavelength. In line with the assays in MDA-MB-231, we found that pVEC has no significant inhibition. We also verified that pVEC-ASO4 produces similar results in HT29 at the highest applied concentration of 500 nM, resulting in 26.35% inhibition. However, at the lower concentrations of 250 nM and 100 nM, we observed lower cell viability than in MDA-MB 231—84.2% and 90.72%, respectively. The pVEC-ASO3 demonstrates an equal cytotoxic effect at the highest concentration in both tested tumor cell lines with the UBA2 gene (Figure S8).

**Figure 8:**
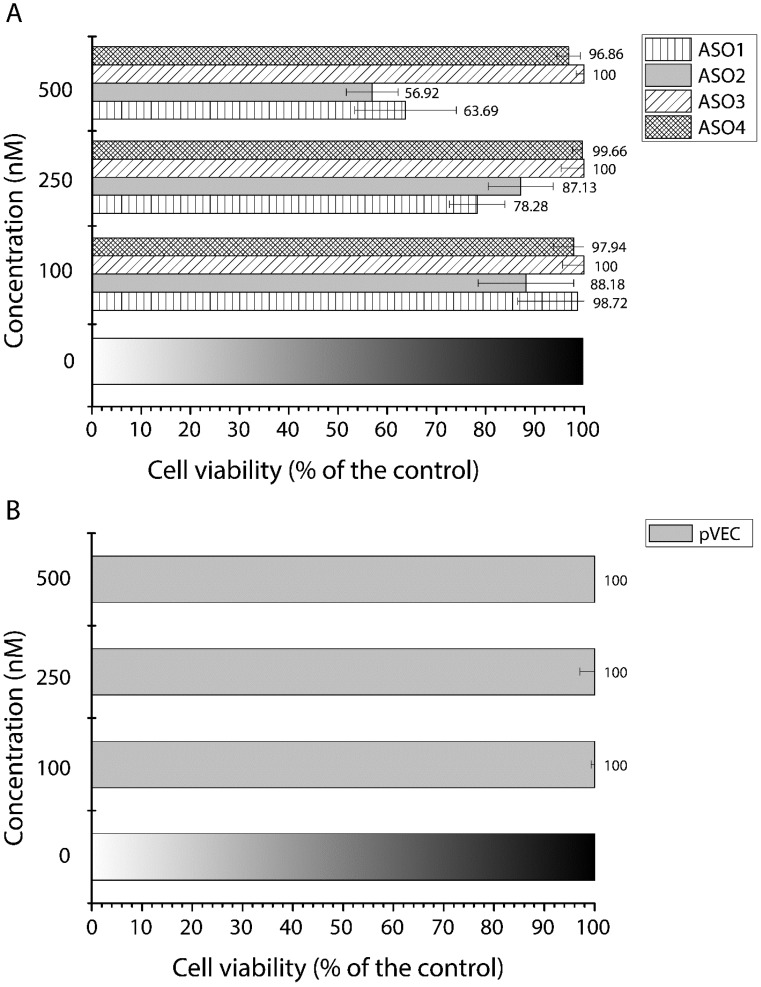
MTT viability assays of the non-tumor fibroblast Lep3 cell line were measured at 540/620 nm. Results are expressed as the percentage of the cell viability relative to the control. Data are presented as the mean ± S.D. after exposure for 72 h to different concentrations of 4 AOSs, including pVEC-ASO1, pVEC-ASO2, pVEC-ASO3, pVEC-ASO4 (**A**), and the CPP pVEC (**B**).

The final MTT assays were performed in non-tumor human fibroblast cell line Lep3 (Figure 8). The experiment was conducted to measure the cell toxicity and cell proliferation of all the designed pVEC-ASOs. To easily compare the new data with the previous MTT assays, we used concentrations of 100 nM, 250 nM, and 500 nM of the tested pVEC-ASOs and pVEC. Among all pVEC-ASOs, one demonstrated cytotoxicity of 11.82% (pVEC-ASO2), two demonstrated cell viability of 97.94% and above, and one revealed no inhibition (pVEC-ASO3) at the lowest concentration (Figure 8A). The increased concentration of 250 nM did not influence the cytotoxic effect of pVEC-ASO2 or the lack of it in cells treated with pVEC-ASO3 and pVEC-ASO4. However, pVEC-ASO1 increased its cytotoxic activity by 20%. At the highest concentration of 500 nM, cells treated with ASO3 demonstrated no signs of cytotoxicity. Lep3 cells treated with pVEC-ASO4 had the second-highest cell viability of approximately 96.9%.

Meanwhile, the cells treated with pVEC-ASO2 demonstrated the lowest viability of 56.9%. Based on the observed results, we calculated an MIC_50_ = 580.32 nM for pVEC-ASO2. By comparing the latest findings with the results in the MDA-MB-231 cell line, ASO3 demonstrates a cytotoxic effect in nearly half the targeted cancer cells and no adverse impact on non-tumor cells Lep3 treated with the highest concentration of 500 nM. Meanwhile, pVEC-ASO1 had a similar cytotoxic effect in healthy and cancerous cells, and pVEC-ASO2 had an even higher cytotoxic effect in the non-tumorous cells. Comparing the results in all three cell lines for pVEC-ASO4 demonstrated higher toxicity in MDA-MB-231 and HT29, with approximately similar values and almost no toxicity in the Lep3 cell line.

## 4. Discussion

In the last decade, antisense oligonucleotide technology has shown various examples of the successful modulation or inhibition of targeted RNAs. Regarding effecting modifications for in vitro and in vivo therapy, the gaptamer ASO is among the most preferred modifications for pVEC-ASOs. Therefore, we designed and tested chimeric antisense oligonucleotides, which combine first- and second-generation modifications. Instead of using only thiol modifications, we also used 2’-O-methyl modifications in the last three bases (pVEC-ASO1 and pVEC-ASO2), the last four bases (pVEC-ASO3), or the last base (pVEC-ASO4) on both ends of the nucleic sequence. That reduces to a minimum the number of thiol-modified nucleotide bases proven to be accountable for non-specific binding to proteins and, consequently, toxicity.

Meanwhile, the remaining PS modifications in the central part of the nucleic sequence are significant enough to activate RNase H cleavage. Hence, we obtain optimal functionality with decreased cell toxicity. Bioinformatics analyses are always needed when choosing the correct ASO sequence since some might still provide evidence of off-target toxicity in the treated organism.

The pVEC-ASO1 and pVEC-ASO2 have only partial hybridization to the guanylate kinase mRNA. They have either weak or no inhibition in *S. aureus*. At the same time, they both demonstrated high toxicity levels in normal and cancer cells at their highest concentration. Since they possess varying but high similarity with essential sequences in the human genome (Figures S2A and S2B), they activate RNase H hybridization by partially hybridizing with human RNAs. It is possible that pVEC-ASO1, which demonstrates similar cytotoxicity cells at the maximum concentration in both cancers (38.07%) and good health (36.31%), is partially hybridizing with either one or both of the two ncRNAs (Figure S2B), such as ncRNA 2778 (LINC02778) and LOC105372520 ncRNA (LOC105372520).

Meanwhile, pVEC-ASO2 exhibits approximately 19% lower cytotoxicity in the human breast cancer line MDA-MB-231 than in the healthy Lep3 cell line. Even though there is a variety among the obtained RefSeq analysis, one seems to be the most probable reason for the displayed results, that is, the 95% similarity (and 95.24% identity) of pVEC-ASO2 with a transcript variant of the BRAF mRNA (Figure S3B), a proto-oncogene that is often mutated in cancer cells (BRAF mutation in thyroid cancer) [45,46,47,48,49]. Thus, we observe a lower cytotoxic effect of pVEC-ASO2 at 500 nM in the MDA-MB-231 cell line (24.56%) than in the Lep3 cell line (43.08%).

On the other hand, pVEC-ASO3 targets UBA2 mRNA, a cancer hallmark pathology in MYC-driven cancer [50,51]. The pVEC-ASO3 demonstrated cytotoxicity in nearly half the MDA-MB-231 cancer cells at 500 nM, while no cytotoxicity was observed in the normal Lep3 cells. That gave us the hope that we might be on the right path with the design of an anti-tumor pVEC-ASO. However, more assays are needed to assess its potential as a safe and effective anti-tumor agent.

ASOs without complementary RNAs in a particular organism should not exhibit off-target hybridization. As such, pVEC-ASO4, designed to have no complementary sequences in bacteria and human genomes, was tested in bacterial cells and human cell lines. It demonstrated the absence of cytotoxicity in *S. aureus* and Lep3 cells, but, interestingly, it affected both cancer cell lines MDA-MB-213 and HT29. We hypothesize that pVEC-ASO4 partially hybridizes with the cancerous RNAs, which leads to their inhibition. It is known that high concentrations of pVEC and pVEC derivates from 4 µM to 20 µM have antibacterial properties.

## 5. Conclusions

Combining PS and 2’-O-methyl modifications allows us to decrease non-specific toxicity and activate the multiple turnover mechanism. However, we can further reduce the number of PS modifications to decrease the non-specific interactions between the ASO and some proteins via a disulfide bond formation. Furthermore, pVEC can penetrate both prokaryotic and eukaryotic cells. Hence, attaching it to the chimeric pVEC-ASOs provides a universal therapeutic agent, inhibiting the mRNAs. Using this general design, we need to change only the nucleic sequence of the ASO to achieve a particular and generally well-tolerated novel therapy. However, despite the current improvements in antisense technology, the risks for non-specific cytotoxicity are still present. There is a clear correlation between the thermodynamic stability of the ASO/target RNA region and the cytotoxic effect of the ASO. Hence, we must employ better bioinformatics analysis to perfect this approach and develop safer and more efficient ASO-based therapeutics.

## Figures and Tables

**Table 1:** Antisense oligonucleotides are used. Lowercase “_1_” stands for “2’-O-CH_3_” modification of the oligomers, and the lowercase “_2_” stands for PS modification of the oligomers.

No	Name	ASO Sequence (5′-3′)	Size (nt)	Molecular Mass (Da)
1	pVEC-ASO1	pVEC-G_1_A_1_U_1_T_2_T_2_T_2_G_2_C_2_T_2_T_2_C_2_T_2_T_2_T_2_A_2_C_2_C_2_T_2_A_2_A_1_U_1_U_1_	22	7906
2	pVEC-ASO2	pVEC-C_1_A_1_A_1_C_2_T_2_T_2_C_2_T_2_T_2_T_2_T_2_C_2_T_2_T_2_G_2_C_2_T_2_T_2_C_2_A_1_U_1_U_1_	22	7856
3	pVEC-ASO3	pVEC-G_1_A_1_U_1_C_1_A_2_A_2_T_2_C_2_A_2_G_2_G_2_T_2_C_2_G_2_A_2_T_2_G_2_T_2_G_2_G_1_G_1_A_1_G_1_	23	8490
4	pVEC-ASO4	pVEC-U_1_A_2_C_2_G_2_C_2_T_2_C_2_G_2_G_2_A_2_C_1_	11	4362

## Data Availability

All data are contained in the article or supplementary materials.

## Supplementary Materials

The following supporting information can be downloaded at: https://www.mdpi.com/article/10.3390/antibiotics13020122/s1, Figure S1. A partition structure of the folding of the mRNA, encoding guanylate kinase in *S. aureus* (strain ATCC 25 923). The model structure is obtained by the RNAfold web server version 2.4.8. Figure S2. Partition structure of the folding of the mRNA, encoding Homo sapiens UBA2 gene. Figure S3. RNA Reference Sequence (RefSeq) analysis for similarities of ASO1’s target with bacterial and human organisms in the GeneBank database. Figure S4. RNA Reference Sequence (RefSeq) analysis for similarities of ASO2’s target with bacterial and human organisms in the GeneBank database. Figure S5. RNA Reference Sequence (RefSeq) analysis for similarities of ASO3’s target with bacterial and human organisms in the GeneBank database. Figure S6. RNA Reference Sequence (RefSeq) analyzes similarities of ASO4’s hypothetical target with all organisms in the GeneBank database. Figure S7. Growth curve of *S. aureus* (strain ATCC 25 923) cultured in LB medium. Figure S8. MTT viability assay of the colorectal adenocarcinoma HT29 cell line was measured at 540/620 nm.
