# Supplementary material for: General and Specific Cytotoxicity of Chimeric Antisense Oligonucleotides in Bacterial Cells and Human Cell Lines
Source: Antibiotics (Basel). 2024 Jan 26;13(2):122. doi: 10.3390/antibiotics13020122 (PMC10885958; doi:10.3390/antibiotics13020122)
Supplement: Supplementary file 1 [file antibiotics-13-00122-s001.zip › antibiotics-2828871-supplementary.pdf]

Supplementary Material

General and specific cytotoxicity of chimeric antisense oligonucleotides in bacterial cells and human cell lines

Katya B. Popova and Robert Penchovsky*

Laboratory of Synthetic Biology and Bioinformatics, Faculty of Biology,

Sofia University “St. Kliment Ohridski”, Sofia, Bulgaria

*To whom the correspondence should be addressed:

Laboratory of Synthetic Biology and Bioinformatics,

Faculty of Biology,

Sofia University “St. Kliment Ohridski”,

8 Dragan Tzankov Blvd.,

1164 Sofia, Bulgaria

Office Phone: +35928167340

E-mail: robert.penchovsky@hotmail.com

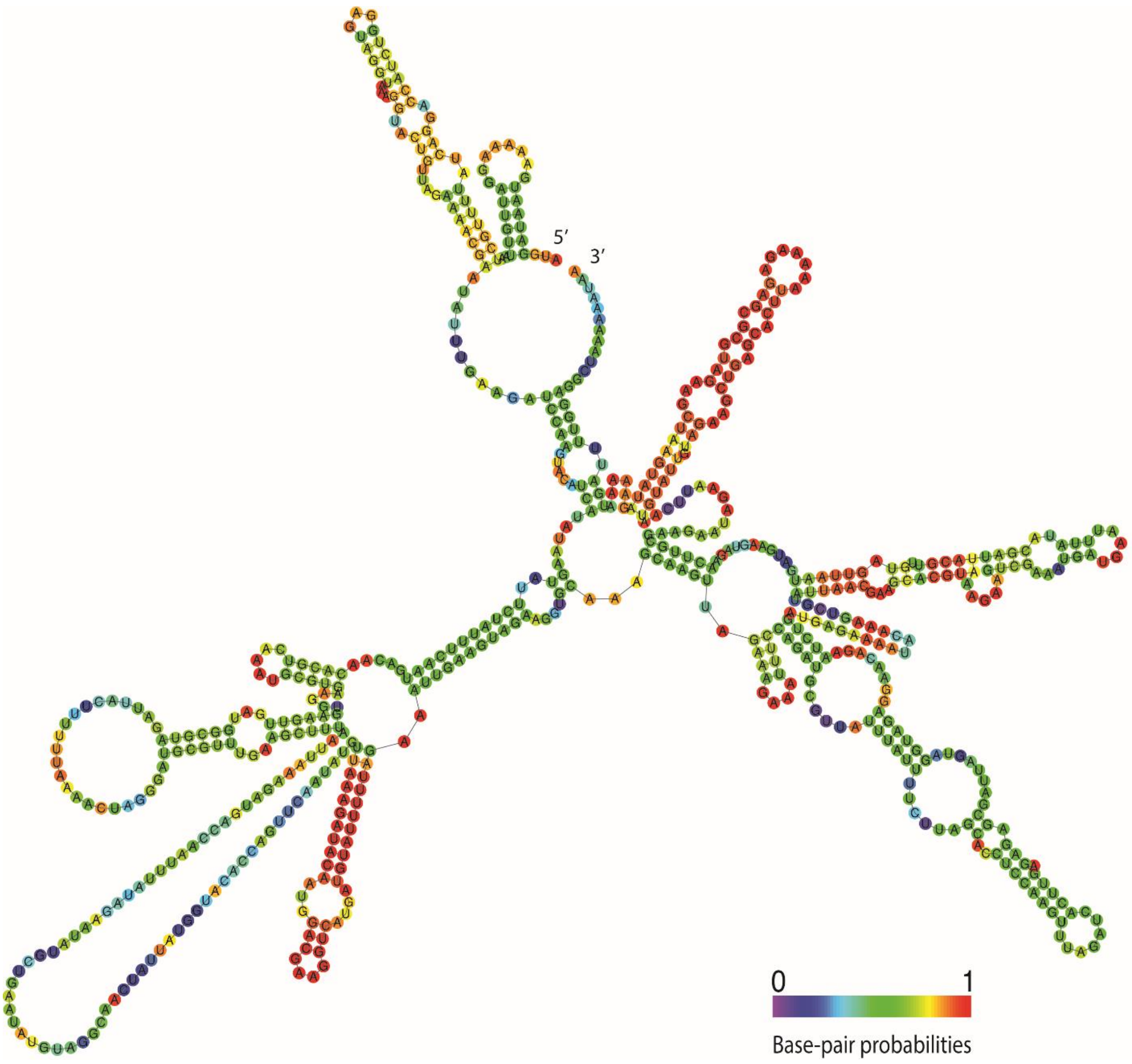

Figure S1. A partition structure of the folding of the mRNA, encoding guanylate kinase in *S. aureus* (strain ATCC 25 923). The model structure is obtained by the RNAfold web server version 2.4.8.

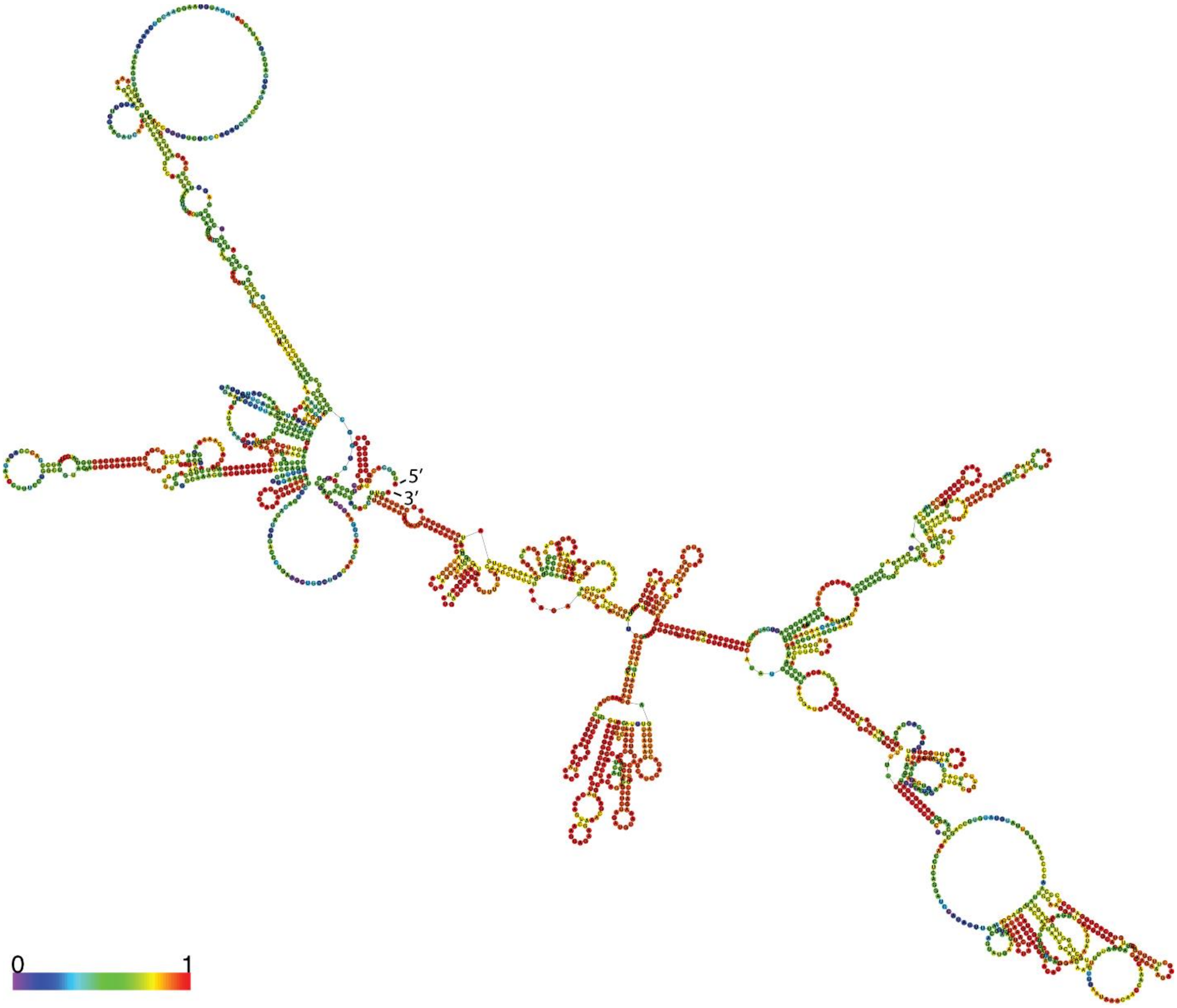

Figure S2. Partition structure of the folding of the mRNA, encoding Homo sapiens UBA2 gene. The model structure is obtained by the RNAfold web server version 2.4.8.

RNA Reference Sequence (RefSeq) was performed for ASO1's target (5'-AAUUAGGUAAAGAAGCAAAAUC) in the GeneBank database to compare it with the curated data in both human and bacterial organisms. The blast results demonstrate the absence of full 100% query coverage in other bacterial organisms with the targeted sequence in the guanylate kinase region in *S. aureus*. Due to varying mismatches, the highest % of similarity is 68%, and it is observed as demonstrated in **Figure S5A**:

- *Prolinoborus fasciculus* (syn. Aquaspirillum fasciculus)
- *Legionella lansingensis*

The same analysis was performed for similarities in the human organism. The results demonstrated a maximum % query coverage of 90% with the targeted sequence in the predicted ncRNA 2778 (LINC02778), followed by 81% in transcript variant X3 in LOC105372520 ncRNA (LOC105372520).

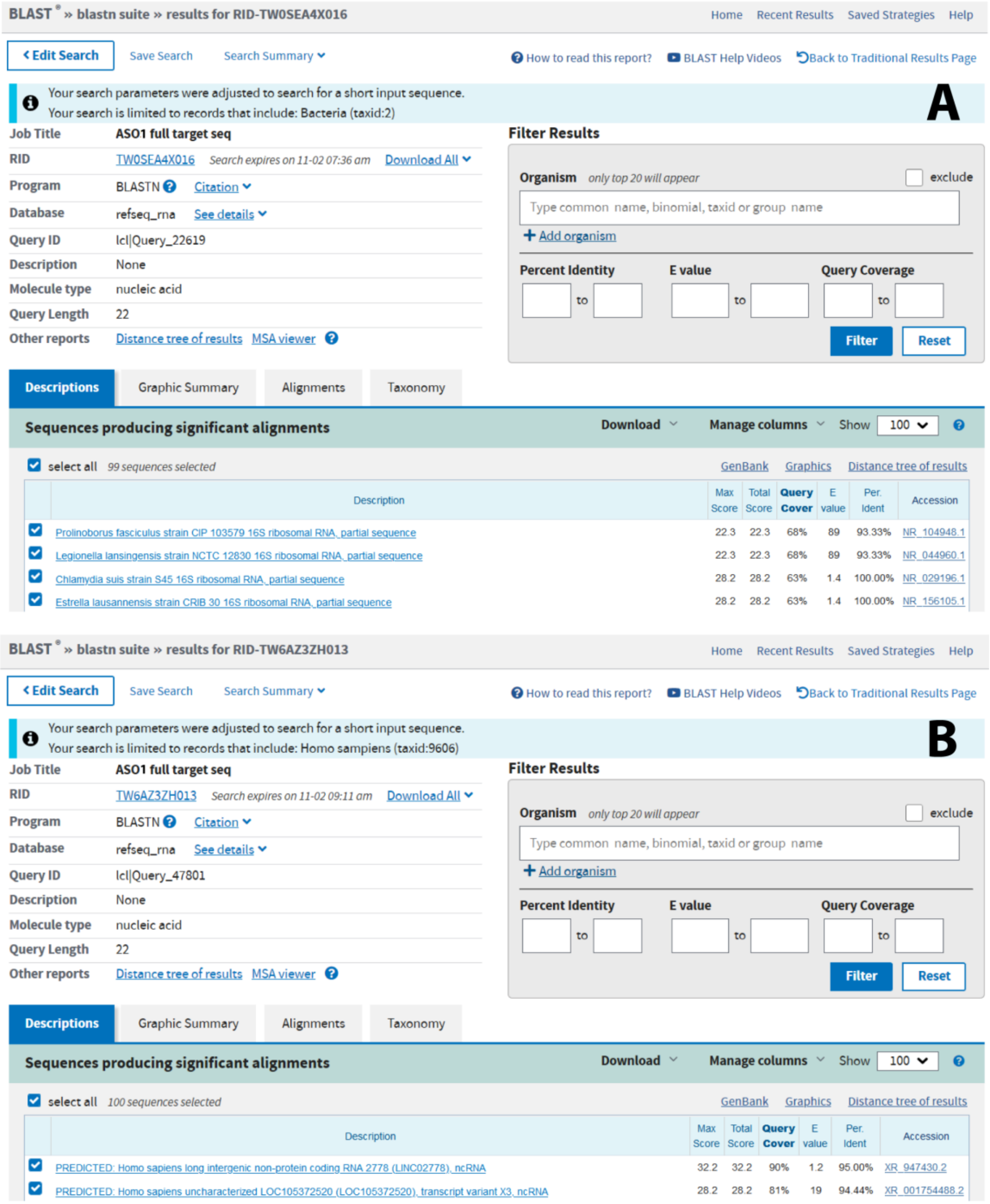

Figure S3. RNA Reference Sequence (RefSeq) analysis for similarities of ASO1's target with bacterial and human organisms in the GeneBank database. **(A)** RefSeq results for similarities of ASO1's target with bacterial microorganisms. **(B)** RefSeq for similarities of ASO1's target within the human organism.

RNA Reference Sequence (RefSeq) was performed for ASO2's target (5'-AATGAAGCAAGAAAAGAAGTTG) in the GeneBank database to compare it with the

curated data in both human and bacterial organisms. The BLAST results demonstrate the absence of full 100% query coverage in other bacterial organisms with the targeted sequence in the guanylate kinase region in *S. aureus*. Due to varying mismatches, the highest % of similarity is 86%, which is observed in the Gram-negative *Candidatus blochmannia*, as demonstrated in Figure S5A**.**

The same analysis was performed for similarities in the human organism. The results demonstrated a maximum % query coverage of 72% between the targeted sequence when there are no gaps (100% identity) and the following:

- 3 transcript variants of the Rho GTPase activating protein 30 mRNA (1, 3, and 4);
- 4 predicted transcript variants of the Rho GTPase activating protein 30 mRNA (X1, X2, X3, and X4);
- 2 predicted transcript variants of uncharacterized LOC105377508 ncPHK (1 and 2).

However, if we allow aligning the results with some gaps or mismatches instead of a 100% identity score, we have several hits that might explain our experimental data. They are in transcription variant of the proto-oncogene BRAF (95% query cover, 95.24% identity), purine-rich element binding protein B (PURB) mRNA (90% query cover, 95% identity), and transcription variants of the center (CNTLN) mRNA (90% query cover, 95% identity). There were also several more predicted variants of those targets among the results.

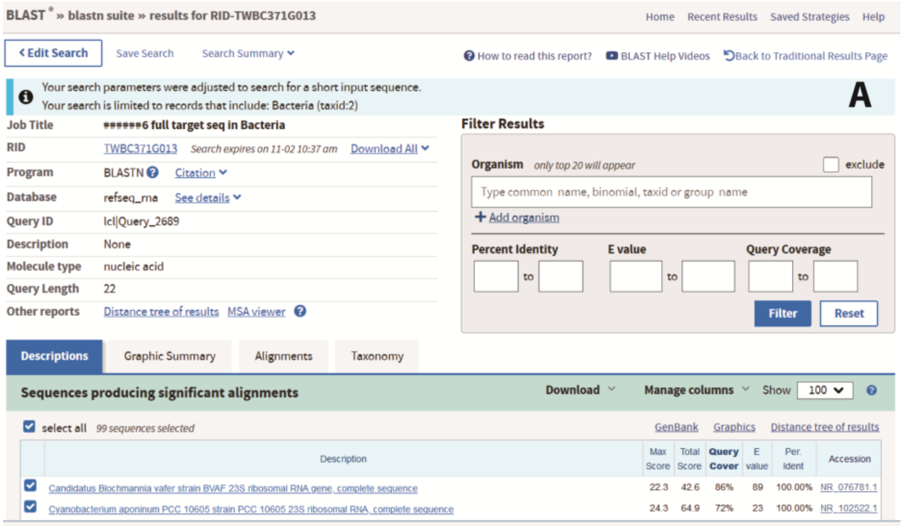

	Description	Max Score	Total Score	Query Cover	E value	Per. Ident	Accession
☑	Candidatus Blochmannia vafer strain BVAF 23S ribosomal RNA gene, complete sequence	22.3	42.6	86%	89	100.00%	NR_076781.1
☑	Cyanobacterium aponinum PCC 10605 strain PCC 10605 23S ribosomal RNA, complete sequence	24.3	64.9	72%	23	100.00%	NR_102522.1

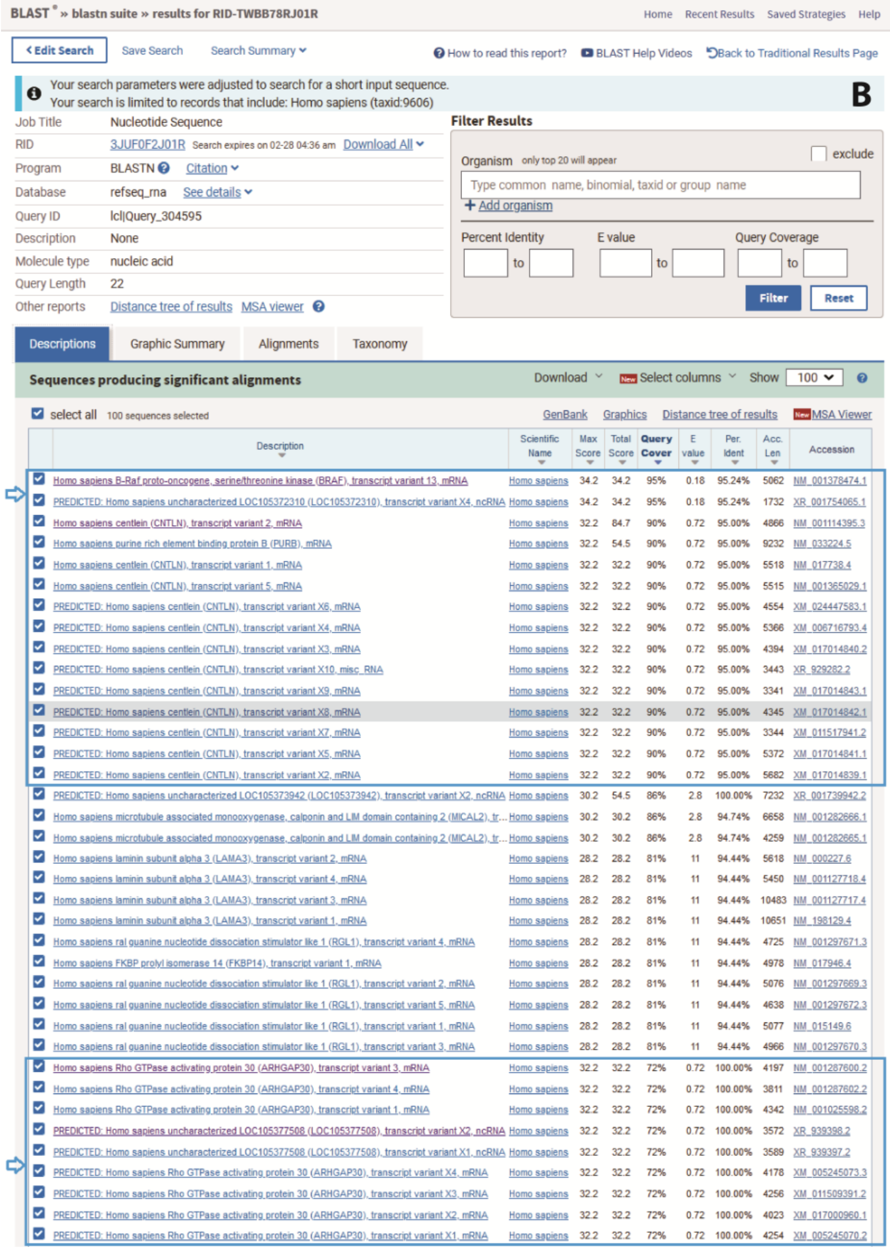

	Description	Scientific Name	Max Score	Total Score	Query Cover	E value	Per. Ident	Acc. Len	Accession
☑	Homo sapiens B-Raf proto-oncogene, serine/threonine kinase (BRAF), transcript variant 13, mRNA	Homo sapiens	34.2	34.2	95%	0.18	95.24%	5062	NM_001378474.1
☑	PREDICTED: Homo sapiens uncharacterized LOC105372310 (LOC105372310), transcript variant X4, ncRNA	Homo sapiens	34.2	34.2	95%	0.18	95.24%	1732	XR_001754065.1
☑	Homo sapiens centlein (CNTLN), transcript variant 2, mRNA	Homo sapiens	32.2	84.7	90%	0.72	95.00%	4866	NM_001114395.3
☑	Homo sapiens purine rich element binding protein B (PURB), mRNA	Homo sapiens	32.2	54.5	90%	0.72	95.00%	9232	NM_033224.5
☑	Homo sapiens centlein (CNTLN), transcript variant 1, mRNA	Homo sapiens	32.2	32.2	90%	0.72	95.00%	5518	NM_017738.4
☑	Homo sapiens centlein (CNTLN), transcript variant 5, mRNA	Homo sapiens	32.2	32.2	90%	0.72	95.00%	5515	NM_001365029.1
☑	PREDICTED: Homo sapiens centlein (CNTLN), transcript variant X6, mRNA	Homo sapiens	32.2	32.2	90%	0.72	95.00%	4554	XM_024447583.1
☑	PREDICTED: Homo sapiens centlein (CNTLN), transcript variant X4, mRNA	Homo sapiens	32.2	32.2	90%	0.72	95.00%	5366	XM_006716793.4
☑	PREDICTED: Homo sapiens centlein (CNTLN), transcript variant X3, mRNA	Homo sapiens	32.2	32.2	90%	0.72	95.00%	4394	XM_017014840.2
☑	PREDICTED: Homo sapiens centlein (CNTLN), transcript variant X10, misc_RNA	Homo sapiens	32.2	32.2	90%	0.72	95.00%	3443	XR_929282.2
☑	PREDICTED: Homo sapiens centlein (CNTLN), transcript variant X9, mRNA	Homo sapiens	32.2	32.2	90%	0.72	95.00%	3341	XM_017014843.1
☑	PREDICTED: Homo sapiens centlein (CNTLN), transcript variant X8, mRNA	Homo sapiens	32.2	32.2	90%	0.72	95.00%	4345	XM_017014842.1
☑	PREDICTED: Homo sapiens centlein (CNTLN), transcript variant X7, mRNA	Homo sapiens	32.2	32.2	90%	0.72	95.00%	3344	XM_011517941.2
☑	PREDICTED: Homo sapiens centlein (CNTLN), transcript variant X5, mRNA	Homo sapiens	32.2	32.2	90%	0.72	95.00%	5372	XM_017014841.1
☑	PREDICTED: Homo sapiens centlein (CNTLN), transcript variant X2, mRNA	Homo sapiens	32.2	32.2	90%	0.72	95.00%	5682	XM_017014839.1
☑	PREDICTED: Homo sapiens uncharacterized LOC105373942 (LOC105373942), transcript variant X2, ncRNA	Homo sapiens	30.2	54.5	86%	2.8	100.00%	7232	XR_001739942.2
☑	Homo sapiens microtubule associated monooxygenase, calponin and LIM domain containing 2 (MICAL2), tr...	Homo sapiens	30.2	30.2	86%	2.8	94.74%	6658	NM_001282666.1
☑	Homo sapiens microtubule associated monooxygenase, calponin and LIM domain containing 2 (MICAL2), tr...	Homo sapiens	30.2	30.2	86%	2.8	94.74%	4259	NM_001282665.1
☑	Homo sapiens laminin subunit alpha 3 (LAMA3), transcript variant 2, mRNA	Homo sapiens	28.2	28.2	81%	11	94.44%	5618	NM_000227.6
☑	Homo sapiens laminin subunit alpha 3 (LAMA3), transcript variant 4, mRNA	Homo sapiens	28.2	28.2	81%	11	94.44%	5450	NM_001127718.4
☑	Homo sapiens laminin subunit alpha 3 (LAMA3), transcript variant 3, mRNA	Homo sapiens	28.2	28.2	81%	11	94.44%	10483	NM_001127717.4
☑	Homo sapiens laminin subunit alpha 3 (LAMA3), transcript variant 1, mRNA	Homo sapiens	28.2	28.2	81%	11	94.44%	10651	NM_198129.4
☑	Homo sapiens ral guanine nucleotide dissociation stimulator like 1 (RGL1), transcript variant 4, mRNA	Homo sapiens	28.2	28.2	81%	11	94.44%	4725	NM_001297671.3
☑	Homo sapiens FKBP prolyl isomerase 14 (FKBP14), transcript variant 1, mRNA	Homo sapiens	28.2	28.2	81%	11	94.44%	4978	NM_017946.4
☑	Homo sapiens ral guanine nucleotide dissociation stimulator like 1 (RGL1), transcript variant 2, mRNA	Homo sapiens	28.2	28.2	81%	11	94.44%	5076	NM_001297669.3
☑	Homo sapiens ral guanine nucleotide dissociation stimulator like 1 (RGL1), transcript variant 5, mRNA	Homo sapiens	28.2	28.2	81%	11	94.44%	4638	NM_001297672.3
☑	Homo sapiens ral guanine nucleotide dissociation stimulator like 1 (RGL1), transcript variant 1, mRNA	Homo sapiens	28.2	28.2	81%	11	94.44%	5077	NM_015149.6
☑	Homo sapiens ral guanine nucleotide dissociation stimulator like 1 (RGL1), transcript variant 3, mRNA	Homo sapiens	28.2	28.2	81%	11	94.44%	4966	NM_001297670.3
☑	Homo sapiens Rho GTPase activating protein 30 (ARHGAP30), transcript variant 3, mRNA	Homo sapiens	32.2	32.2	72%	0.72	100.00%	4197	NM_001287600.2
☑	Homo sapiens Rho GTPase activating protein 30 (ARHGAP30), transcript variant 4, mRNA	Homo sapiens	32.2	32.2	72%	0.72	100.00%	3811	NM_001287602.2
☑	Homo sapiens Rho GTPase activating protein 30 (ARHGAP30), transcript variant 1, mRNA	Homo sapiens	32.2	32.2	72%	0.72	100.00%	4342	NM_001025598.2
☑	PREDICTED: Homo sapiens uncharacterized LOC105377508 (LOC105377508), transcript variant X2, ncRNA	Homo sapiens	32.2	32.2	72%	0.72	100.00%	3572	XR_939398.2
☑	PREDICTED: Homo sapiens uncharacterized LOC105377508 (LOC105377508), transcript variant X1, ncRNA	Homo sapiens	32.2	32.2	72%	0.72	100.00%	3589	XR_939397.2
☑	PREDICTED: Homo sapiens Rho GTPase activating protein 30 (ARHGAP30), transcript variant X4, mRNA	Homo sapiens	32.2	32.2	72%	0.72	100.00%	4178	XM_005245073.3
☑	PREDICTED: Homo sapiens Rho GTPase activating protein 30 (ARHGAP30), transcript variant X3, mRNA	Homo sapiens	32.2	32.2	72%	0.72	100.00%	4256	XM_011509391.2
☑	PREDICTED: Homo sapiens Rho GTPase activating protein 30 (ARHGAP30), transcript variant X2, mRNA	Homo sapiens	32.2	32.2	72%	0.72	100.00%	4023	XM_017000960.1
☑	PREDICTED: Homo sapiens Rho GTPase activating protein 30 (ARHGAP30), transcript variant X1, mRNA	Homo sapiens	32.2	32.2	72%	0.72	100.00%	4254	XM_005245070.2

Figure S4. RNA Reference Sequence (RefSeq) analysis for similarities of ASO2's target with bacterial and human organisms in the GeneBank database. (A) RefSeq results for similarities of ASO2's target with bacterial microorganisms. **(B)** RefSeq for similarities of ASO2's target within the human organism. With blue lines and arrows marked, the two types of maximum % hits are either query cover but lack of whole identity or the complete 100% identity with lower query cover than the targeted sequence.

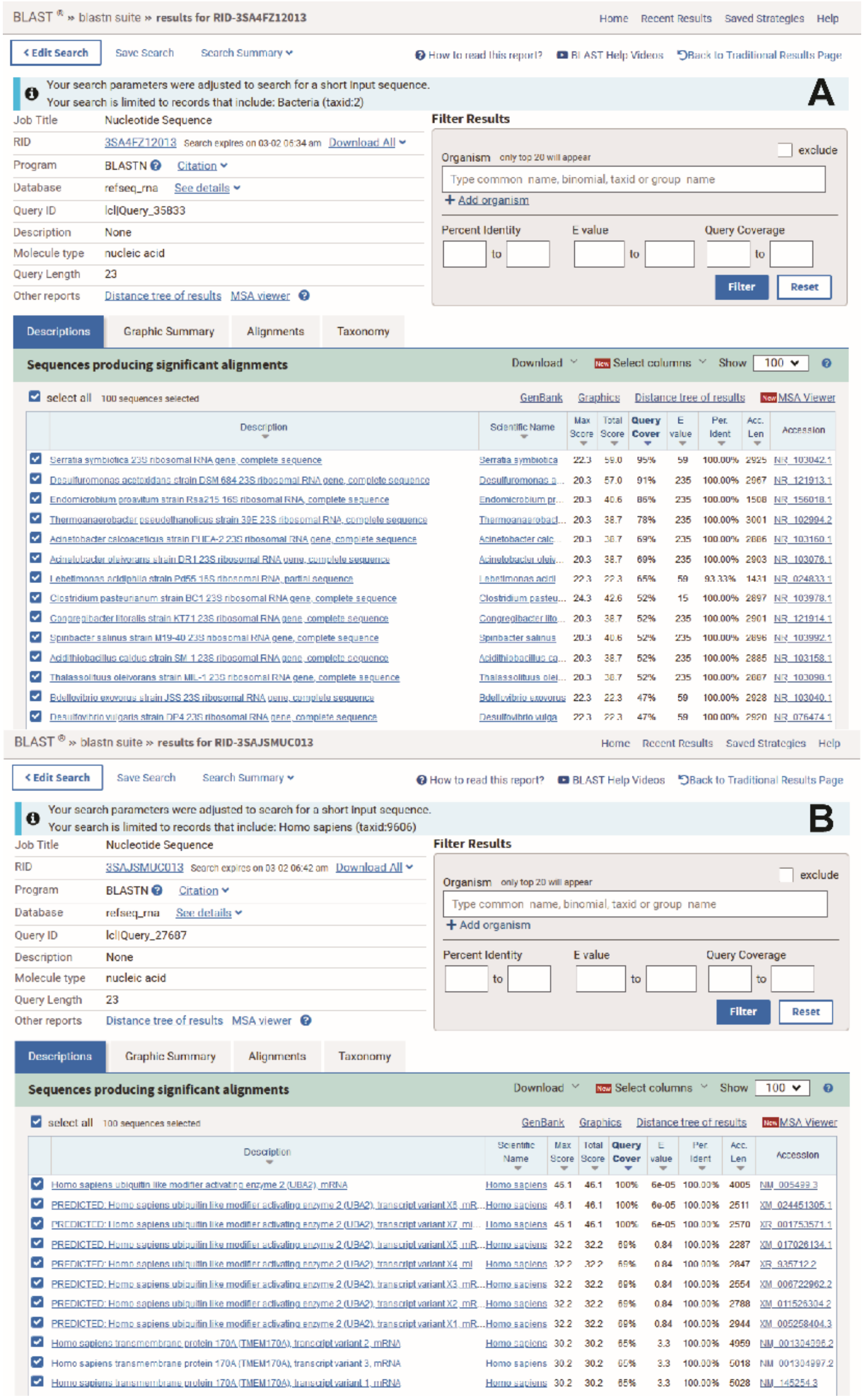

Figure S5. RNA Reference Sequence (RefSeq) analysis for similarities of ASO3's target with bacterial and human organisms in the GeneBank database. (A) RefSeq results for similarities of ASO3's target with bacterial microorganisms. **(B)** RefSeq for similarities of ASO3's target within the human organism.

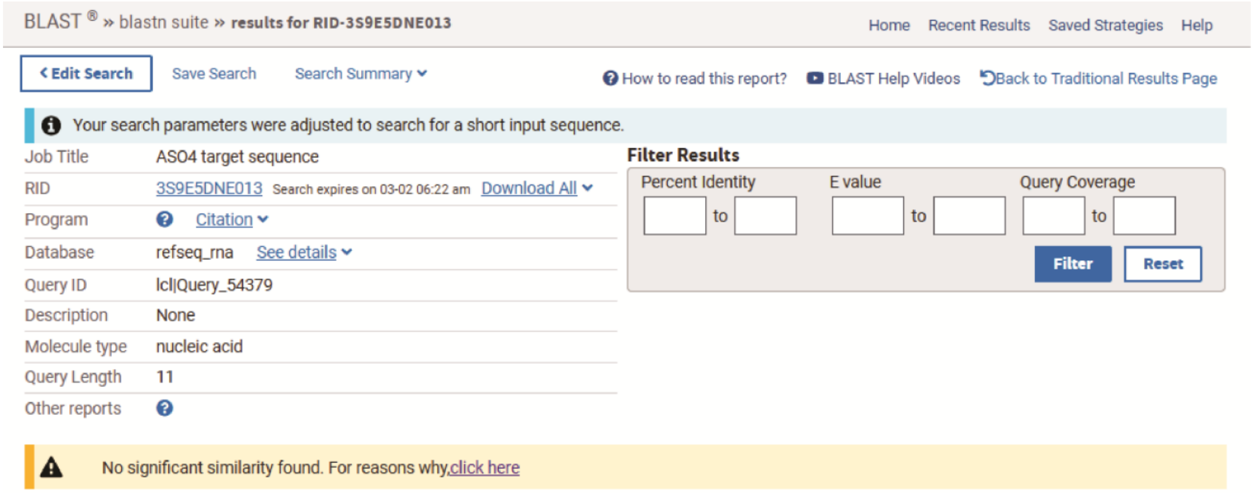

Figure S6. RNA Reference Sequence (RefSeq) analyzes similarities of ASO4's hypothetical target with all organisms in the GeneBank database.

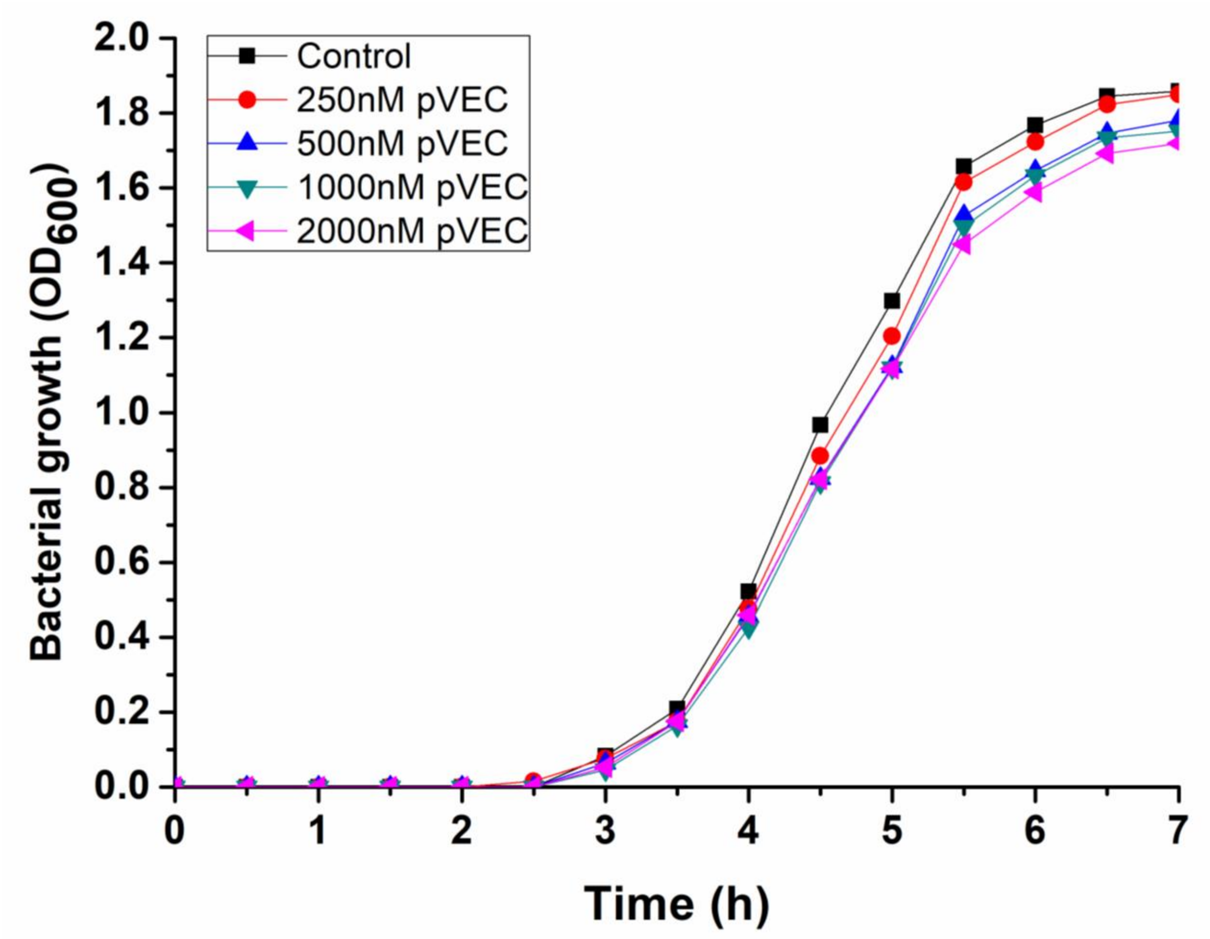

Figure S7. Growth curve of *S. aureus* (strain ATCC 25 923) cultured in LB medium. Bacteria were treated with different concentrations of pVEC: 250nM (red line), 500nM (blue line), 1000nM (green line), and 2000nM (purple line). Treated bacteria were compared to the control with untreated bacteria (black line). The measurements were assessed in cuvettes by spectrophotometer at 600nm wavelength.

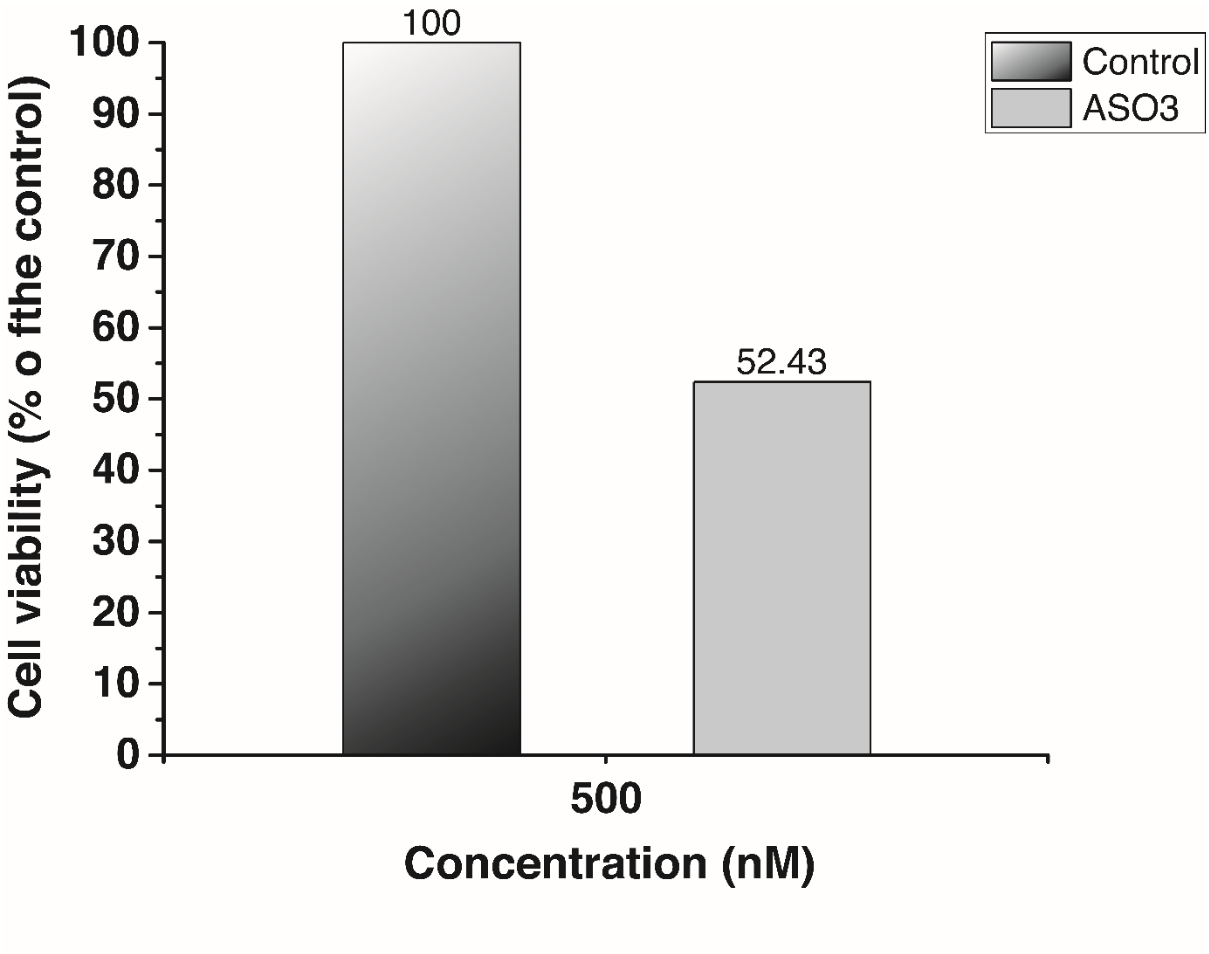

Figure S8. MTT viability assay of the colorectal adenocarcinoma HT29 cell line was measured at 540/620nm. Results are expressed as the percentage of the cell viability relative to the control. The 500nM pVEC-ASO3 is tested after exposure for 72h.
